# The ethical challenges of palliative care from the perspectives of pediatricians: A qualitative study in Iran

**DOI:** 10.3389/fped.2022.928476

**Published:** 2022-08-29

**Authors:** Farzaneh Zahedi, Maliheh Kadivar, Leila Khanali Mojen, Mahsa Asadabadi, Saleheh Tajalli, Mahnaz Ilkhani, Salman Barasteh, Maryam Elahikhah, Bagher Larijani

**Affiliations:** ^1^Endocrinology and Metabolism Research Center, Endocrinology and Metabolism Clinical Sciences Institute, Tehran University of Medical Sciences, Tehran, Iran; ^2^Division of Neonatology, Department of Pediatrics, Children's Medical Center, Tehran University of Medical Sciences, Tehran, Iran; ^3^School of Nursing and Midwifery, Shahid Beheshti University of Medical Sciences, Tehran, Iran; ^4^Pediatric Congenital Hematologic Disorders Research Center, Research Institute for Children's Health, Shahid Beheshti University of Medical Sciences, Tehran, Iran; ^5^Nursing Care Research Center, School of Nursing and Midwifery, Iran University of Medical Science, Tehran, Iran; ^6^School of Nursing and Midwifery, Shahid Beheshti University of Medical Sciences, Tehran, Iran; ^7^Health Management Research Center, Baqiyatallah University of Medical Sciences, Tehran, Iran; ^8^Nursing Faculty, Baqiyatallah University of Medical Sciences, Tehran, Iran; ^9^Students Research Committee, Baqiyatallah University of Medical Sciences, Tehran, Iran; ^10^Medical Ethics and History of Medicine Research Centre, Tehran University of Medical Sciences, Tehran, Iran; ^11^Endocrinology and Metabolism Research Center, Endocrinology and Metabolism Clinical Sciences Institute, Tehran University of Medical Sciences, Tehran, Iran

**Keywords:** ethical challenge, palliative care, ethical issues, pediatrician, qualitative study, end of life, life-limiting

## Abstract

**Background:**

Adherence to ethical principles is a requirement for palliative care delivery to children and a main concern of healthcare providers. Physicians usually face ethical challenges during their daily practice in hospitals and need adequate skills and the ability to identify and manage them. This study sought to explore the ethical challenges of palliative care from the perspectives of pediatricians.

**Methods:**

This qualitative study was conducted between April and July 2019 using the content analysis approach. Participants were fifteen pediatric medical residents, specialists, and subspecialists purposively recruited from pediatric hospitals in Tehran, Iran. Data were collected using in-depth semi-structured interviews and were analyzed using Graneheim and Lundman's approach to conventional content analysis. Trustworthiness was ensured through the four criteria proposed by Guba and Lincoln.

**Results:**

Participants' experiences of the ethical challenges of palliative care for children were grouped into two main categories, namely “bewilderment in dealing with children and their families” (with two subcategories) and “conflicts in decision making” (with three subcategories). The final five subcategories were: (a) inability to effectively communicate with children and their families, (b) inability to tell the truth about the disease, (c) physician-parent conflicts, (d) parent-child conflicts, and (e) physician-physician conflicts.

**Conclusion:**

The main ethical challenges of palliative care from the perspectives of Iranian pediatricians are the inability to effectively communicate with children and their families, the inability to tell them the truth, and the inability to manage physician-parent, parent-child, and physician-physician conflicts. Identification and management of these challenges may help improve the quality of pediatric palliative care in Iran. Further studies are needed to confirm these findings in other settings.

## Introduction

The prevalence of life-limiting conditions is increasing among children worldwide. This prevalence is 18.5–32 cases per 10,000 persons among children aged more than 1 year and 127.3 cases per 10,000 persons among children aged <1 year (1–3). According to World Health Organization, about 21 million children may need palliative care worldwide each year (4). The heavy disease burden and complex care-related needs of children with these conditions highlight the great need for Pediatric palliative care (1).

Pediatric palliative care is active physical, mental, and psychological care for children with life-limiting conditions and support for their families from the point of diagnosis and irrespective of treatments. Pediatric palliative care is designed and provided by assessing and attempting to reduce physical, mental, and social distresses at hospital, community, and home levels using a team approach involving healthcare providers, families, and community resources. It is an ideal care delivery approach for patients with chronic or incurable conditions which can fulfill the physical, mental, spiritual, and social needs of afflicted children and their families and improve their quality of life (5). Palliative care is considered an ethical responsibility of healthcare providers toward patients with life-limiting conditions (6).

Children are in a period of rapid growth and development, have many different needs, and have varying levels of understanding about phenomena. Therefore, the ethical challenges (ECs) of palliative care for children are also different from those of adults (4). Previous studies reported a wide range of ECs of palliative care for children. For instance, a study in Taiwan showed that the three main ECs of palliative care for children were related to truth-telling, palliative care setting, and fulfillment of children's basic needs (7). These challenges in India were related to end-of-life concerns and resuscitation (8). In Jordan, physicians reported mothers' inability to decide on end-of-life care as the main EC of palliative care for children, while palliative care-related ECs in Turkey were related to children's decision-making ability and informed consent for decision making (9, 10). Studies in Iran also reported that the ECs of palliative care were related to interaction with families, their end-of-life concerns, lack of guidelines for do-not-resuscitate order, concerns with child autonomy, and ambiguities in the principles of ethics (11, 12). The wide variety of these challenges across different studies may be due to differences in sociocultural contexts.

The above-mentioned studies show that ECs of Pediatric palliative care are mainly related to patient autonomy in decision making. However, previous studies, particularly those in Iran, rarely addressed other main ethical principles, namely beneficence, non-maleficence, and justice. Moreover, there is limited information about medical specialists' perspectives on Pediatric palliative care-related ECs. Schofield et al. showed that there is a lack of data about ethical issues in low- and middle-income countries (13). Therefore, this study was conducted to address these gaps. The aim of the study was to explore the ECs of Pediatric palliative care from the perspectives of Iranian pediatricians.

## Materials and methods

### Study design and participant selection

This qualitative study was conducted in a 4-month period (April–July 2019) using the content analysis approach. The study setting was four specialized university hospitals for children in Tehran, Iran. Participants were 15 pediatricians (residents, specialists, and subspecialists) who were purposively recruited. Inclusion criteria were specialty pediatric medicine, a work experience of at least 2 years, work in children's specialty hospitals, and agreement for participation. Exclusion criteria were voluntary withdrawal from the study or moving to another hospital during the study. No pediatricians refused to participate or withdrew after giving their consent. The study report uses the consolidated criteria for reporting qualitative research (COREQ) checklist (14) (Supplementary Table 1).

### Data collection

Data were collected using in-depth semi-structured interviews held in a quiet place in the participants' workplace. The interviews were conducted by the corresponding author (LKM), a 48-year-old woman who is a faculty member of School of Nursing & Midwifery of Shahid Beheshti University of Medical Scinces and fellow of palliative care. The time and the place of the interviews were arranged according to participants' preferences. Field notes were not taken during the interviews. The researcher introduced himself and the research objectives, obtained permission to record conversations and the possibility of referral to validate the data, and then asked the participants to describe their experiences. Initially, general questions were used to get familiar with participants, create a friendly atmosphere, and assess their information about palliative care. Then, interviews were guided using questions related to the study aim such as, “Can you explain your experiences of care delivery to a child with an incurable disease?” and “What challenges did you experience in care delivery to a child with an incurable disease?”. The interviewer attempted to collect more in-depth data using pointed questions such as, “What do you mean by this?”, “What happened after that?”, and “Can you provide clearer explanations about this?”. The questionnaire used in semi-structured interviews is reported in Supplementary Table 2. All interviews were recorded (with interviwees' permission) using a digital voice recorder and immediately transcribed word by word. Data collection was kept on up to the point of data saturation achieved with thirteen participants. The length of the interviews was fifty minutes, on average.

### Data analysis

In order to analyze qualitative data, Graneheim and Lundman method was used (15). The coding process was conducted by removing duplicate codes and merging similar codes according to constant comparative analysis. The interviews were transcribed by ST and LKh; ST and FZ read them several times to gain an understanding of the entire interview. Following that, we identified the meaning units and the primary codes. The text was divided into semantic units that were compressed. The compressed meaning units were abstracted and labeled with a primary code. Afterward, similar primary codes were condensed and merged into subcategories, and the main categories were extracted. For encoding and data management, MAXQDA software version 10 was used (16). During the data analysis process, software output was taken several times, and the codes, subcategories, and categories were provided to the entire research team. Then changes were made according to the opinions and consensus of the research team. An example of data analysis is shown in Table 1.

**Table 1:** Example of data analysis.

**Categories**	**Subcategories**	**Basic codes**	**Quotation**
Bewilderment in dealing with children and their families	Inability to tell the truth about the disease	Hesitation in telling the truth to child	“…when the child is still conscious, I don't know if the child should be told the truth or not and explain that her illness is serious and may lead to death...”

### Trustworthiness

Trustworthiness was ensured through the four criteria proposed by Guba and Lincoln, namely credibility, dependability, confirmability, and transferability (17) (Table 2).

**Table 2:** Guba and Lincoln criteria.

**Criteria**	**Sub-criterion**
Credibility	Prolonged engagement with the data
	Member checking
	Allocating adequate time to data collection and analysis
Dependability	Data triangulation
	External peer checking
Confirmability	Clear descriptions about all steps of the study in order to track the process of the study
Transferability	SAMPLING with maximum variation with regard to participants' age, occupation, and educational level

### Ethical considerations

The current research was a part of a comprehensive study carried out to find the shortcomings and educational needs in the field of Pediatric palliative care, and to design and compile an educational booklet for pediatricians. This study has the approval of the Ethics Committee of Endocrinology and Metabolism Research Institute (EMRI) of the Tehran University of Medical Sciences, Tehran, Iran (IR.TUMS.EMRI.REC.1398.007). Before interviews, participants were ensured about data confidentiality and their freedom to withdraw from the study, and then their written informed consent was obtained.

## Results

### Participants

Nine female (60%) and six male (40%) pediatricians participated in the study. The means of their age and work experience were 43.33 (±8.93) and 11.26 (±4.98) years, respectively. Table 3 shows the characteristics of study participants.

**Table 3:** Participants' characteristics.

**No**.	**Age (years)**	**Gender**	**Educational level**	**Work experience** **(years)**	**Interview duration** **(minutes)**
1	49	Male	Pediatric fellowship in intensive care	10	45
2	47	Male	Pediatric fellow in intensive care	12	43
3	42	Male	Pediatric fellow in intensive care	8	64
4	41	Female	Pediatric oncologist	12	40
5	52	Female	Pediatric oncologist	20	35
6	58	Male	Pediatric oncologist	25	47
7	40	Female	Neonatal subspecialist	9	60
8	39	Female	Neonatal subspecialist	10	75
9	48	Female	General pediatrician	13	46
10	40	Female	General pediatrician	9	37
11	37	Male	Pediatric rheumatologist	9	50
12	42	Female	Pediatric rheumatologist	10	47
13	38	Male	Pediatric pulmonologist	9	42
14	40	Female	Pediatric oncologist	7	68
15	37	Female	General pediatrician	6	53

In total, 785 final codes were developed, which were categorized into five subcategories and the two main categories of “bewilderment in dealing with children and their families” and “conflicts in decision making” (Table 4).

**Table 4:** Categories, subcategories, and examples of quotations.

**Subcategory**	**Quotation**
**Category: bewilderment in dealing with children and their families**	
Inability to effectively communicate with children and their families	“*We are not familiar with the necessary psychological skills for establishing effective communication with patients* *with chronic and incurable diseases. We don't know when and how we can communicate with children and* *families who are experiencing a crisis” (Participant no. 6)*.
Inability to tell the truth about the disease	“*Affliction of a child with an incurable disease is like a severe earthquake which ruins all wishes of a family. We* *can't make a right decision about how to deal with such a family. We can neither give false hope to these families* *nor tell them the truth about the disease. We feel pangs of conscience in these situations and don't know which* *decision is the best and complies with the ethical principles. We don't really have the necessary skills for managing* *these situations” (Participant no. 11). “I don't know whether I should tell the truth to children who are in the last* *days of life and are still conscious. I know that if I tell the truth to these patients and then something wrong* *happens to them, their families would definitely attribute that to my truth-telling practice. Do we have adequate* *legal support in these situations?” (Participant no. 2)*.
**Category: conflicts in decision making**	
Physician-parent conflicts	“*When we have a child with poor prognosis, it is very difficult to inform the family that there is no effective* *treatment for the child and to persuade them to take the child home. They usually insist on hospitalization and* *performance of any possible intervention. In these situations, we really become bewildered and don't know what* *the best decision is and how important the benefit of a child who can't defend himself/herself is” (Participant no. 4)*.
Parent-child conflicts	“*For using some types of mechanical ventilation such as non-invasive ventilation, we need to involve the afflicted* *children in decision making in order to improve their collaboration. We have had some patients who did not accept* *such treatments and noted that they preferred death, while their families insisted on treatments. Who is the* *ultimate decision maker in these situations? Can we respect the decisions of a legally competent child when we* *know that taking an action or no action is harmful for him/her?” (Participant no. 13). “Conflicts between children* *and families are serious, though families make the final decisions without involving their children in decision* *making in almost all situations. This is a cultural problem. What should we do? Taking ethics into account or* *respecting cultural beliefs?” (Participant no. 12)*.
Physician-physician conflicts	“*We had a terminally-ill patient in our ward who had severe neutropenia, thrombocytopenia, and anemia and* *was intubated. I really didn't know what decision I could make; discontinuation of all treatments or their* *continuation despite the child's severe suffering. There was no consensus among colleagues mainly due to their fear* *over legal prosecution” (Participant no. 4). “Our major challenge is legal concerns about resuscitation. Medical* *ethics and medical law should be consistent. We need clear formal protocols and guidelines which legally support* *us” (Participant no. 5)*.

### Bewilderment in dealing with children and their families

According to the participants, affliction of a child by a chronic or incurable disease leads to a serious crisis and psychological breakdown for families. Hearing the news of diagnosing an incurable disease is difficult and unpleasant for parents and often causes them negative psychological reactions such as fear, guilt, anger, shock, and denial. Immediately after getting informed about the diagnosis, most families start seeking information in order to find a ray of hope to deny the disease. Participants noted that in these situations, they became bewildered and could not effectively communicate with families and adhere to the ethical principle of veracity. This category had two subcategories, namely “*inability to effectively communicate with children and their families*” and “*inability to tell the truth about the disease*”.

#### Inability to effectively communicate with children and their families

Participants noted that effective communication, which is based on veracity, confidence, commitment, and secrecy, helps establish therapeutic relationships, improves care quality, and facilitates clinical decision-making. According to the participants, patients and the families' knowledge and cultural beliefs and values are determining factors in effective communication. They noted that conflicts in cultural beliefs can interfere with the establishment of effective communication with patients. They also introduced their own lack of knowledge about communication skills as a major challenge in establishing effective communication with patients and families. Moreover, they acknowledged that ineffective communication with patients and families can result in an atmosphere of distrust and reported families' uncertainty as a major barrier to effective communication.

#### Inability to tell the truth about the disease

Participants noted that sometimes family members in Iran do not want to tell their ill children about their diseases, while children may seek information. In such conflicting conditions, physicians become bewildered whether to tell the truth to children or not. Our participants reported their limited truth-telling ability as one of the major ECs of palliative care for children and noted that factors such as uncertainty, children's developmental age, and psychological distress including anxiety and grief contributed to their ECs.

### Conflicts in decision making

According to the participants, the best decisions for palliative care are those collectively made by physicians, families, and ill children. Besides the ethical principle of autonomy, other principles, i.e., beneficence, non-maleficence, and justice, should be considered in making decisions for children with incurable diseases. Participants divided conflicts in decision-making for children with incurable diseases into three main types. The first type of conflicts was related to conflicts between parents and physicians, which happened when patients had poor prognosis and hence, physicians attempted to persuade parents to accept the discontinuation of some medical interventions due to the lack of the necessary resources. The second type of conflicts was related to conflicts between parents and children, which occurred when ill children had the necessary legal competence for decision making, but families independently made decisions without considering children's preferences. The third type of conflicts was conflicts among physicians which were related to the discontinuation of treatments and resuscitation, and occurred in the absence of clear guidelines and adequate legal support for them.

#### Physician-parent conflicts

Participants noted that in Iran, families make the ultimate decisions for their ill children due to their own concerns, pangs of conscience, and attempt to help children. In order to save their children's lives, families try to find the best treatment options and may even ignore palliative care and insist on futile invasive interventions. Participants reported that these conditions caused them uncertainties and ECs. Moreover, the lack of some healthcare resources, such as home-based palliative care services, reduced participants' ability to make accurate decisions. They noted that some families may consent to take their ill children to home while lack of home care services caused them uncertainties and made decision-making difficult.

#### Parent-child conflicts

Participants noted that due to sociocultural beliefs, families do not usually value children's preferences and hence, physicians cannot decide whether parents' preferences or child's preferences should be taken into account during the process of treatment.

#### Physician-physician conflicts

Participants noted that major ECs of palliative care for children were related to decision-making for patients with terminal conditions. They noted that their major concerns in these situations were related to resuscitation or non-resuscitation and continuation or discontinuation of futile treatments. They highlighted the lack of clear protocols and guidelines, lack of professional skills, fear of legal prosecution, and cultural beliefs as the main factors contributing to such ECs.

## Discussion

### Main findings

This qualitative study aimed to explore the ECs of Pediatric palliative care from the perspectives of pediatricians in Iran. Findings showed that the two main ECs of palliative care were “bewilderment in dealing with children and their families” and “conflicts in decision making”.

### Interpretation and comparison with previous literature

One of the main categories of the study was bewilderment in dealing with children and their families, which implies physicians' inability to manage the crisis related to the diagnosis of an incurable disease in the family. Inability to effectively communicate with children and their families was a subcategory. Affliction of a child with an incurable disease is a serious crisis in the family that can result in reactions such as stress, concern, anger, and denial. Therefore, a key component of palliative care is to establish effective communication with parents in order to reduce their stress and concerns. However, our participants reported that they had limited communication skills and were unable to effectively communicate with children with incurable disease and their families. In line with this finding, a previous study in Iran found that more than half of the medical students did not have the necessary skills for effective communication (18) and this is associated with ineffective symptom management, impaired quality of life, medical errors, and inaccurate perception of the conditions of ill children and their families (19, 20). Therefore, studies highlighted the necessity of providing healthcare providers with quality and systematic education about effective communication skills (19–22).

Our participants' inability to establish effective communication with patients and their families reduced their ability to manage families' uncertainties and problems. Uncertainty is a common problem among the parents of children with chronic incurable diseases (23). Uncertain parents continuously experience states of hope and despair. Some of them may avoid seeking information in order to keep their hope alive, while some others may seek information to find the best treatment options to save their children's lives which in turn may cause them despair. In these situations, physicians' sincere communication can be helpful (24).

Findings also showed the inability to tell the truth to children and their families as another ECs of palliative care. According to the ethical principle of veracity, healthcare providers should tell the truth to patients and their families. Koch and Jones showed that families expected physicians to tell them the truth (25). According to our participants, factors such as children's developmental age, family culture, and dynamicity, and psychological distresses can make truth-telling difficult. In certain communities, the ethical principle of non-maleficence is considered more important than the ethical principle of autonomy, and the whole family is considered more important than individual family members; therefore, truth concealment is common in these communities (26, 27). On the other hand, families may decide not to tell the truth to their ill children in order to protect them against psychological distresses, while children's better understanding of the truth can promote their coping and collaboration (28). In line with our findings, a former study in Iran reported the concealment of the truth from ill children (29), and pediatric residents in another study in Iran considered giving bad news to ill children and their families as a major EC and reported that they needed education in this area (11). One of the main concerns of our participants respecting truth-telling was their fear of its associated legal consequences. They noted that physicians in Iran face problems and conflicts in telling the truth to patients. Actually, telling the truth is a culturally related issue and each country may have its own practice (30, 31). However, legal system should be in harmony with ethical standards. As a case in point, the family may sue the physician for telling the truth to the patient and claim that the breaking bad news has resulted in the patient's stress, hopelessness, depression, and anxiety. This challenge should catch the attention of Iranian health authorities.

The second main category of the study was conflicts in decision-making. This category was related to conflicts in clinical decision making among decision-makers. One of the subcategories of this category was physician-parent conflicts. Palliative care-related decisions are collaboratively made by physicians, families, and children (32), mainly based on the ethical principle of beneficence and hence, may result in conflicts regarding the ethical principle of autonomy (10). Our participants noted that omitting futile treatments has always been a major challenge in most countries, creating problems in physicians' decision-making. They highlighted that physician-parent conflicts were mainly due to parents' insistence on using futile treatments or omitting essential treatments due to socioeconomic problems. Parents may insist on using unnecessary treatments to manage their sense of guilt, denial, the stigma of being a bad parent, and their fear of hastening their child's death (33). In fact, parents' insistence on the continuation of invasive interventions or discontinuation of treatments is due to their oscillation between hope and despair which affects their decisions and results in decision-making based on their own needs and conditions rather than the best possible options for the child. Such a type of decision-making contradicts the ethical principles of beneficence and justice (34, 35).

Successful attainment of the goals of palliative care depends on the available resources for service delivery. Some participants expressed concerns over the discontinuation of unnecessary treatments and patient transfer from hospital to home due to the lack of the necessary resources. This contradicts the ethical principles of respect, dignity, and justice (36). In line with this finding, a former study reported the lack of necessary resources for home care as a major EC in care delivery in India (8). Similarly, home care for children with cancer is still considered a dream in Iran (37). Moon et al. reported the lack of necessary resources as a major EC in care delivery to patients in outpatient settings (38).

We also found that another cause of physician-parent conflicts was omitting the necessary treatments due to parents' socioeconomic problems. Such omission contradicts the ethical principle of beneficence. In agreement with this finding, a former study reported financial problems as a factor contributing to parents' unwise decisions (32). Although recent healthcare reforms and the provision of free medical services in hospitals in Iran have significantly reduced challenges related to families' financial problems, this may still affect parents' decisions. When omitting treatments is harmful to patients, clinical ethics committees can be a useful strategy for collective decision-making (36). According to our participants, the members of such committees can be forensic physicians, legal authorities, medical ethics specialists, and attending physicians.

Another subcategory of the conflicts in decision-making was parent-child conflicts. Study participants reported unclear regulations, lack of knowledge, cultural context, and family relationships as factors contributing to the omission of children from decision-making. Such ignorance contradicts the ethical principle of autonomy. A former study in Iran also reported that pediatric residents had challenges managing parent-child conflicts due to their lack of knowledge in this area (11). Rassouli et al. reported that the involvement of children in clinical decision-making in Iran is still associated with many challenges (22). However, families in Eeastern countries often make the ultimate decisions without the meaningful involvement of their children (30).

Findings also revealed physician-physician conflicts as the other subcategory of the conflicts in decision-making category. Decision-making about the continuation or discontinuation of treatments for children with terminal conditions is a source of major ECs for physicians. Although the American Academy of Pediatrics confirms the omission of heavy treatments for terminally ill children in order to improve their quality of life, there are still controversies in this area due to factors such as physicians' different cultural and religious beliefs, emotional backgrounds, lack of knowledge, and fear of legal problems (38, 39). Similarly, Brock et al. reported culture, religion, and sociodemographic characteristics such as age, ethnicity, and language as major factors affecting end-of-life decisions (40).

Most people in Iran are Muslim and have religious beliefs about death and dying. For example, they believe that life and death are in the hands of God. Such beliefs directly affect end-of-life decisions, such as the decision for treatment discontinuation or non-resuscitation. Although the “do not resuscitate” code is being used since many years ago in many Western countries, it is still illegal in many Islamic countries if it equates to life termination (12). Some Islamic scholars, however, make a distinction between avoidance of prolonging the process of death and active ending of life; and permit the healthcare team to make appropriate decisions through consultation with the family (41, 42). It is a rather new concept in most Asian countries. For instance, the “do not resuscitate” code was approved in India in 2014 (43). In Iran, there is no clear ethical and clinical guideline for the “do not resuscitate” code, and decisions in this area are mainly made and verbally announced by physicians without informing patients and their families. Therefore, culturally appropriate guidelines in this area are needed for the context of Iran (12).

## Limitations

This study has several limitations. First was the difficulty in recruiting an adequate number of participants that would allow data saturation. This restriction led to all the participants being selected from Tehran, the capital of Iran. Second, our findings may reflect the situation in Iran, but may not apply to other settings and sociocultural contexts.The possibility that participants in the study did not remember ECs was another limitation. The most important limitation of the present study was that most participants were unfamiliar with pediatric palliative care delivery as a holistic discipline. Therefore, we had some problems with performing interviews about related ethical issues. The problem was minimized by providing the interviewees with some starting explanations.

## Conclusions

Pediatric palliative care for terminally-ill children is associated with different ECs, which vary according to the immediate sociocultural context. The findings of the present study can increase medical specialists' sensitivity to ethical issues and highlight the necessity of assessing and fulfilling their palliative care-related needs. Our findings emphasized the ethical aspects of palliative services, and identified ethical problems that may be prevalent in many countries in our region or worldwide. This study also may convince policy-makers and health authorities to pay more attention to building the necessary infrastructure for strengthening team-working in pediatric palliative care services in Iran. We encourage the education of pediatricians through short or long courses, or even professional fellowship programs, to allow them to be familiar with various physical and psychological issues in pediatric palliative care, and play a leading role in ethical decision-making in everyday medical practice. Further studies are needed to confirm these findings in other settings.

## Author's note

The office of World Health Organization (WHO) in Tehran provided financial support of the research. However, the opinions discussed in the paper does not necessarily represent the views of WHO.

## Data availability statement

The raw data supporting the conclusions of this article will be made available by the first author, without undue reservation.

## Author contributions

FZ and MK conceived and designed the study with help of BL. LK, MA, and ST developed the theory in practice and carried out the interviews. LK, MI, SB, and ME analyzed the data. LK wrote the draft of the paper which was edited and approved by FZ, MK, MA, ST, MI, SB, ME, and BL. Critical revision was done by MK, BL, and FZ. All authors contributed to the article and approved the submitted version.

## Funding

The office of World Health Organization funded the project (as a part of APW contract of 2019-878455-0).

## Conflict of interest

The authors declare that the research was conducted in the absence of any commercial or financial relationships that could be construed as a potential conflict of interest.

## Publisher's note

All claims expressed in this article are solely those of the authors and do not necessarily represent those of their affiliated organizations, or those of the publisher, the editors and the reviewers. Any product that may be evaluated in this article, or claim that may be made by its manufacturer, is not guaranteed or endorsed by the publisher.
