# Supplementary material for: The ethical challenges of palliative care from the perspectives of pediatricians: A qualitative study in Iran
Source: Front Pediatr. 2022 Aug 29;10:928476. doi: 10.3389/fped.2022.928476 (PMC9464941; doi:10.3389/fped.2022.928476)
Supplement: Supplementary file 2 [file Table_2.DOC]

**Interview schedule**

**1. Orientation**

The aim of our study was to explore the ethical challenges of palliative care from the perspectives of pediatricians. To do this, your consent to participate in the study is required, although you can interrupt the interview and withdraw from the study at any time. I have to record the interview. Then I transcribe them. Your name will not be mentioned in the report or article. First, fill out the informed consent form with demographic information including age, sex, years of experience, educational status, and marital status.

**2. Primary questions**

*Can you explain your experiences of care delivery to a child with a life-threating condition?*

The next questions are based on the participants' experiences. Examples:

- How many life-threating or chronic patients did you treat?
- What stress did you experience while caring for these patients?
- How was your experience of caring for dying children?

**3. Main question**

*What ethical challenges did you experience in care delivery to a child with a life-threating condition?*

**4. Probing question**

Based on the participants' experiences probing questions were asked. Examples:

- What is your experience of providing palliative care to these children and their families?
- Based on your experience, what are the dimensions of ethical challenges in caring for these children and their families?
- Did you have any conflict for treating?
- How was your experience for telling true to these children and their family?

**5. Terminal phase**

The researcher's questions and discussions are over, please let me know if you have any other points or questions.

**Thank you for your participation in this study.**
